# Promises and challenges of a decentralized CAR T-cell manufacturing model

**DOI:** 10.3389/frtra.2023.1238535

**Published:** 2023-09-05

**Authors:** Manan Shah, Ashley Krull, Lynn Odonnell, Marcos J. de Lima, Evandro Bezerra

**Affiliations:** ^1^ Department of Hematology, the James Cancer Hospital and Solove Research Institute, Ohio State University, Columbus, OH, United States; ^2^ Department of Cell Therapy Manufacturing and Engineering, the James Cancer Hospital and Solove Research Institute, Ohio State University, Columbus, OH, United States; ^3^ Department of Hematology, Cellular Therapy Lab, the James Cancer Hospital and Solove Research Institute, Ohio State University, Columbus, OH, United States; ^4^ Department of Hematology, The James Cancer Hospital and Solove Research Institute, Ohio State University, Columbus, OH, United States

**Keywords:** decentralized, CAR T, leukapheresis, chimeric, lentivirus

## Abstract

Autologous chimeric antigen receptor-modified T-cell (CAR T) products have demonstrated un-precedent efficacy in treating many relapsed/refractory B-cell and plasma cell malignancies, leading to multiple commercial products now in routine clinical use. These positive responses to CAR T therapy have spurred biotech and big pharma companies to evaluate innovative production methods to increase patient access while maintaining adequate quality control and profitability. Autologous cellular therapies are, by definition, manufactured as single patient batches, and demand has soared for manufacturing facilities compliant with current Good Manufacturing Practice (cGMP) regulations. The use of a centralized production model is straining finite resources even in developed countries in North America and the European Union, and patient access is not feasible for most of the developing world. The idea of having a more uniform availability of these cell therapy products promoted the concept of point-of-care (POC) manufacturing or decentralized *in-house* production. While this strategy can potentially decrease the cost of manufacturing, the challenge comes in maintaining the same quality as currently available centrally manufactured products due to the lack of standardized manufacturing techniques amongst institutions. However, academic medical institutions and biotech companies alike have forged ahead innovating and adopting new technologies to launch clinical trials of CAR T products produced exclusively in-house. Here we discuss POC production of CAR T products.

## Limitations of current autologous chimeric antigen receptor T-cell manufacture model

Autologous chimeric antigen receptor-modified T-cell (CAR T) therapy has revolutionized the management of relapsed/refractory B-cell and plasma-cell malignancies. There are six different FDA approved autologous CAR T products manufactured by four different pharmaceutical companies (Table 1) (1–12). There are also more than 200 active CAR T clinical trials in the United States to optimize CAR T therapy efficacy, mitigate toxicities and broaden disease indications to additional hematologic malignancies, solid tumors, and even non-malignant diseases (13–18) The use of autologous CAR T therapy is expected to grow exponentially, but this growth will likely exacerbate two major current limitations of CAR T therapy: patient access and financial burden to the healthcare system (19–22).

**Table 1:** Commercially produced CAR-T cell products.

Product	Pharmaceutical company	Target/Year	Indications	FDA approved	UK/EU approved
Kymriah (Tisagenlecleucel)	Novartis	CD-19 (2017)	ALL, NHL	Yes	Yes
Yescarta (Axicabtagene)	Kite/Gilead	CD-19 (2017)	NHL, Follicular lymphoma	Yes	Yes
Tecartus (Brexucabtagene)	Kite/Gilead	CD-19 (2020)	ALL, Mantle cell	Yes	Yes
Breyanzi (Lisocabtagene)	BMS	CD-19 (2021)	NHL	Yes	Yes
Abecma (Idecabtagene)	BMS	BCMA (2021)	Multiple Myeloma	Yes	Yes
Carvykti (Ciltacebtegene)	Jansen	BCMA (2022)	Multiple myeloma	Yes	No

Access is a major limitation even in large academic comprehensive cancer centers in the United States, where five out of the six FDA approved CAR T products were developed, and where the current commercial products are centrally manufactured. The access may be limited by the CAR T manufacturing capacity offered by pharmaceutical companies (23–25). Patients often wait up to 3 weeks for a “manufacturing slot”, an allocated date when the company can receive the patient's autologous apheresis product to start manufacturing CAR T cells (26–29).

Another major access limitation is the prolonged manufacturing and release time that frequently ranges from 2 to 4 weeks. Candidates for CAR T therapy usually have aggressive diseases and are heavily pre-treated, and often may decline clinically and become ineligible while waiting to receive treatment (24, 25, 27). Manufacture delays can be attributed to the approved methods used for transduction and expansion of T-cells, mainly based on technology from when the products were initially developed over 10 years ago. Moreover, because of the centralized nature of the process, shipment, and cryopreservation are required adding more time to the clinically significant “vein-to-vein” time (time from leukapheresis to CAR T infusion) (28).

Lastly, the current costs of commercially manufactured CAR T products total nearly $500,000 USD, in addition to other clinical costs with medications, hospitalization, transfusions etc. Such costs present a major burden on the healthcare system of developed countries and, therefore, are prohibitive for most of the population in the developing world (21, 22, 28). There is an unmet need to optimize the current model of CAR T manufacture, so both the “vein-to-vein” time and costs are minimized. To this end, there are two approaches of large interest: use of off-the-shelf allogeneic CAR T and decentralized in-house autologous CAR T manufacture (30–33) Despite the enthusiasm for the development of off-the-shelf allogeneic CAR T seen in multiple ongoing trials, so far their efficacy and persistence have been limited by rejection, on top of significant donor variability leading to significant T-cell fitness differences (31, 34, 35).

Here, we review the decentralized in-house CAR T manufacturing model. We also discuss different platforms available for decentralized in-house manufacturing (Table 2) and the challenges associated with implementation of these procedures.

**Table 2:** Different place-of-care manufacturing.

Author	Production time	Indications	Manufacturing platform
Ortiz-Maldonado et al. (28)	8–11 days. Cryopreserved	Acute lymphoblastic leukemia, non-Hodgkin's lymphoma Chronic lymphocytic leukemia	ARI-0001 (locally produced vector) cells at a dose of 0.4–5 × 10^6^ cells/kg. CliniMACS Prodigy©.
Maschan et al. (32)	8–12 days Fresh cells	Relapsed/refractory pediatric B-cell ALL and adult B-cell NHL. Phase I clinical trials in Moscow (Russia) and Cleveland (USA)	(Lentigen©) CliniMACS Prodigy©.
Palani et al. (30)	12 days	No patients received the product.	(Lentigen©) CliniMACS Prodigy©.
Kedmi et al. (24)	10 days Fresh cells	Adult patients with aggressive B-cell lymphoma.	Locally produced anti-CD19 retrovirus vector with a CD28 costimulatory domain.
Shah et al. (11)	14 days Fresh cells	Adult patients with B cell non-Hodgkin lymphoma or chronic lymphocytic leukemia	(Lentigen©) CAR T cells; CliniMACS Prodigy©.

## General concept of CAR T manufacturing

There are two different logistical approaches to CAR T manufacturing: (1) the standard centralized process where the product is manufactured in cell therapy laboratories controlled by the pharmaceutical company; (2) the decentralized method where CAR T products are manufactured in one or more cell therapy laboratories within the academic healthcare system to support clinical trials and eventually even commercial manufacturing. The outline of various steps involved is mentioned below (1, 3, 4, 5, 6, 12, 13, 15, 20, 24, 32, 36–38).

(1) **Cell collection:** Peripheral blood mononuclear cells (PBMCs) are collected through leukapheresis in an accredited healthcare facility. The cells are transported in a stringent cold chain system either at 2–8°C for fresh products or below −150°C for cryopreserved products (20, 36, 37).
(2) **CAR T manufacturing:** CAR T products are manufactured in accordance with Food and Drug Administration (FDA) current Good Manufacturing Practice (cGMP) regulations, following multiple steps, detailed below (39).
  (a) T-cell enrichment from peripheral blood mononuclear cells (PBMCs) and activation using CD3/CD28 antibodies (5, 6, 12, 40–46).
  (b) Genetic engineering of the T-cells to induce CAR expression, most commonly through transduction with viral vectors (non-viral approaches are under investigation) (1, 3–6, 47–49).
  (c) CAR T expansion and harvesting in the presence of specific cytokines (1, 3–6, 15, 42, 45).
  (d) QC testing and release by the QA unit prior to distribution is critically important and typically takes 2–3 weeks. Fresh CAR T products typically must be infused within 48 h of harvest (19, 20, 50).
(3) **Administration:** After release from centralized manufacture, the cryopreserved product is shipped in temperature-monitored packaging to the healthcare facility where the patient will be treated. Prior to infusion of CAR T products, patients are subjected to a lymphodepleting agent over several days (1, 3–6).

## CAR T manufacturing systems: open/manual vs. closed/automated

Traditionally, small-scale manufacturing procedures utilize open systems in which a bag, tube or culture vessel with cells is open to the environment, albeit in an aseptic manner. Such open systems are almost completely manual and, as such, require multiple highly skilled laboratory scientists and technologists working hands-on for several full days over the culture period. Compared to truly closed systems, open systems are also more susceptible to microbiologic contamination and human error. Product manipulation is conducted in a classified cleanroom. CAR T production also requires co-culture of the stimulated T-cells with a gene vector (frequently a replication deficient viral vector), which also must be handled in a controlled environment that prevents exposure of the technical personnel or the laboratory environment (42, 51–54).

Alternatively, closed/automated systems utilize platform devices (e.g., CliniMACS Prodigy©, Lonza Cocoon© and others) that enable the apheresis product to move through the multiple steps of CAR T manufacture (T-cell isolation, activation, transduction, and CAR T expansion) inside a single use, disposable kit that is not directly opened to the environment. Sample removal and reagent or media addition are accomplished through sterile tubing welders or aseptic access ports, thus maintaining what is known as a “functionally closed system”. These automated systems may be operated in facilities with less stringent air classifications (i.e., ISO 8 or even unclassified space, as opposed to ISO 7) and mean significantly less hands-on time, and more consistent handling during manufacture. These advantages make automated manufacturing devices the preferred method for decentralized CAR T manufacture. It should be noted, however, that currently available devices are used for one CAR T product at a single time. To accommodate large patient volumes, centers would need to have multiple instruments with high price tags and requiring significant bench or floor space and implement well-controlled protocols for optimally timing the ending of one product's manufacture and apheresis collection and initiation of another product's run (13, 15, 23, 24, 30, 32, 38, 55, 56).

A third option is a hybrid semi-closed/semi-automatic system. Here, each individual step is automated, but done in separated devices with minimal manual product handling between steps/modules. This process can be facilitated by a robotic arm to minimize human error (57).

## CAR T manufacturing location: centralized vs. decentralized

### Centralized

All CAR T cell products approved by the FDA are manufactured centrally. The treating physician prescribes a specific FDA approved CAR T product and patient's PBMCs are shipped from the treating healthcare facility to the central manufacturing cell therapy laboratory. Often, pharmaceutical companies have a facility in North America and another in the European Union to supply the two main CAR T markets. After manufacture and cryopreservation, the product is shipped back to the treating healthcare facility (1, 3–6, 12). The main advantage of centralized manufacture is quality standardization that minimizes inter-product variability. The increased oversight and control afforded by a centralized manufacturing model was critical for commercialization of a labor-intense manual process (19, 20, 29). However, due to the personalized nature of autologous CAR T production, it is not possible to deliver a truly uniform CAR T product. There also remain many drawbacks with the centralized model as listed previously: long waiting time, patient access, and financial burden. Centrally manufactured CAR T products often spend more time in the QC/QA processes than in manufacturing (19, 20, 29).

### Decentralized

In the decentralized model, the product is manufactured within or very near the same healthcare system where the patient will be treated. This model minimizes or eliminates the need for cryopreservation and improves timing and potentially costs. For instance, eliminating cryopreservation of the starting material can potentially influence cell quantity and quality, as the recovery and viability of PBMCs is often reduced after freezing and thawing compared with fresh apheresis products (24, 29, 30, 38, 58, 59). In the USA, the decentralized manufacturing model has been restricted to academic centers in the context of clinical trials. Initially, prior to CAR T FDA approvals, heavily manual POC manufacture was used exclusively for early phase, single center clinical trials at a select few institutions. Later, these groups transferred their technology and patents to biotechnology startups and large pharmaceutical companies that led the multi-center studies that adopted centralized manufacture strategies and resulted in the current FDA approvals (51–54).

The rapid development of bioreactors and other technologies is enabling a paradigm shift in which POC production is becoming more accessible. The use of automated closed systems including such as CliniMACS Prodigy© and Cocoon© has drastically reduced the need for clean rooms thereby decreasing the need for expensive infrastructure (15, 32, 60) Importantly, these automated systems comply with federal regulations requiring software that may generate electronic records involved in the manufacture of biologics. These systems can currently accommodate a lentiviral gene vector or non-viral vectors for T-cell transduction, with all subsequent steps through formulation which are conducted within this closed automated unit (61) The final product often is produced in a shorter manufacturing time (commonly 7–10 days vs. 14 days) and at a lower cost compared to centralized manufacturing, although there are not commercially available POC CAR T in the USA. Decentralized manufacturing also removes the risks and costs of transportation and may be infused fresh. The average cost of production of in-house CAR T cells can be as low as $35,000 USD if viral vector is provided by a sponsor or collaborator and is variable between $50,000- $1 million if GMP vector must be purchased by the center (29, 30) This does not include costs required for setup, staffing and maintenance.

## Manufacturing platforms available for POC production

Here, we briefly describe the available bioreactors that provide a functionally closed GMP-compliant cell processing system. Various pros and cons of each platform is discussed in Table 3 (23).

(A) *CliniMACS Prodigy® system-* The CliniMACS cell system was established in 1997 by Miltenyi Biotec for enrichment and depletion of specific cell types using magnetically labeled antibodies. The Prodigy system represents a technology that automates all the necessary steps of CAR T production beyond T cell enrichment, such as activation, transduction, washing, and media feeding in one closed tubing system. An electroporation attachment is now available allowing gene editing capabilities on one device. Though this platform ensures GMP compliance and reduces strict clean room requirements, some steps are still manual and extensive training is required. Nevertheless, this system is commonly used for production in academic medical centers throughout the world (13, 30, 61, 62).
(B) *The Cocoon® platform*- The Cocoon system from Lonza is another closed manufacturing system that is based on a single use transportable cassette. The cassette internalizes all the media and reagents and can maintain the reagents in a temperature-controlled environment, although T cell enrichment currently must be performed on a CliniMACS device. The limitation again is that some steps are still manual, particularly if electroporation is needed (58, 60).
(C) *ekko*™ *acoustic cell processing system*- The ekko™ system from MilliporeSigma is a novel GMP-compliant platform that utilizes acoustophoresis as a method of cell processing and production. This can be utilized for separating TCR-positive from TCR-negative cells and has been used for other therapies like NK cells. The disadvantage of this system is that it has not been widely utilized for CAR T cell production and hence data is limited (62).
(D) *G-Rex® bioreactor M series*- Wilson Wolf's gas permeable rapid membrane technology allows G-Rex flasks to support the production of high cell density products over short culture durations in a familiar tissue culture flask format, but without the large capital investment of fully automated systems. Several different sizes from 2 cm^2^ to 500 cm^2^ make scaling up a classic T-flask method straight-forward. In addition, closed system G-Rex flasks with sterile fluid paths are now available, along with a liquid handling pump, the GatheRex, that simplifies and accelerates media exchange and cell harvesting. A limitation of this system is that manual monitoring of cell density and decision-making about splitting cultures to additional vessels are required quite often during production (62, 63).
(E) Other technologies that are still under review to support widespread POC production include: the ThermoGenesis CAR TXpress™ platform, the Gibco CTS Rotea™, the Terumo Quantum® cell expansion system and the Cytiva perfusion media (62–64).

**Table 3:** Different manufacturing platforms for CAR-T production.

Manufacturing platforms	Advantages	Disadvantages	Clinical application
CliniMACS Prodigy	1. Closed system performing all steps from cell preparation and harvest to final formulation. 2. Fresh or thawed peripheral blood mononuclear cell (PBMC) products can be loaded directly. 3. Extremely flexible platform. 4. High cell output with limited processing time.	1. This system is driven by complex software that requires close monitoring and detailed training of operators. 2. High cost of installation and maintenance.	Used by Maschan and Shah et al. for CAR T production for relapsed/refractory B-cell ALL and Non-Hodgkin's lymphoma (11, 32). Used for in-house, POC CD19/20/22 CAR T production (NCT05418088).
Lonza Cocoon	1. Simple operability as it uses customized cassettes. 2. Can use reagents and multiple stimulating agents from various manufacturers. 3. Lower cost of installation and maintenance.	1. Requires stringent temperature control during processing. 2. Initial steps of T-cell enrichment and transduction remain manual and require utilization of the CliniMACS system. 3. Lower output when compared to CliniMACs.	Used in combination with CliniMACS Prodigy (11, 32). Used in production of CD-19 CAR T production for relapsed non-Hodgkin's lymphoma by Anguille et al. (60).
Wilson Wolf G-Rex (Gas permeable) bioreactor system	1. Simple, cost effective, and practical in its use for production. 2. Manufactures multiple cell types. 3. Cost-effective installation and maintenance.	1. Risk of contamination as some steps need to be performed manually e.g., electroporation and depletion of cells. 2. Higher use of man-power and increased need for training. 3. Lower cell output per unit. Requires multiple units for large-scale processes.	Being evaluated for genetically engineered T-cell expansion to treat human papilloma virus associated cancers (NCT02858310).

## Challenges to implementation of POC production

For all the promises and advancements in POC production, the process of implementing such a manufacturing system includes many challenges that must be overcome, including personnel training, quality management, facilities design, financing, and reagent sourcing.

Automated systems often use vectors including genetically modified lentivirus for transduction of CAR T cells during their production. Limited commercial production of GMP-grade viral vectors presents a universal challenge for all institutions aiming to manufacture CAR T cells. Institutions looking to implement POC manufacturing should establish early partnerships with viral vector suppliers or consider bringing GMP vector production in house to have better control over timelines. Interestingly, vector manufacturing capability is expanding worldwide (19, 20, 65–67). It is important to note that the cost will vary depending on the amount of testing required by the client, the size of the vector batch produced, and shipping requirements. Institutions would also be wise to consider sourcing research-grade vector preparations (often at a cost of less than $50,000) to enable development work and early validation activities before transitioning to a GMP-grade preparation for final validation runs.

POC manufacturing requires a highly skilled technical team with a working knowledge of aseptic techniques, clinical-grade reagents, and a variety of release assays to prove a product is suitable for use in humans. With automated POC platforms, the challenges presented by release tests are often more difficult to overcome than the actual manufacturing. This is further complicated when fresh CAR T products are desired, as a large QC team is required to perform STAT PCR testing of vector copy number, replication competent virus and mycoplasma, STAT flow cytometric testing of percent transduced cells and other cell types, along with other release tests such as endotoxin. Unfortunately, finding, training, and retaining skilled personnel is increasingly becoming a challenge for academic institutions. Creative workforce recruitment and training approaches are needed to expand the workforce to meet the demand. Infrastructure in the form of GMP-compliant lab space, procurement of additional space and external equipment will present a significant cost burden. To help in this effort, newer manufacturing platforms aim to remove this drawback and make operation in a lower-class environment possible (39) In essence, use of a closed production platform outside of a clean room is dependent on the classification of the space and the validation performed by the manufacturing staff demonstrating that the risk of contamination is mitigated to the same degree as if the product were being processed in a clean room environment (e.g., through technologist best practices, aseptic technique, environmental monitoring). Though regulations differ between countries, to our knowledge there are no regulations that prohibit the use of closed production platforms outside of a cleanroom if manufacturing practices are properly validated and documented.

Quality control is one of the greatest challenges to implementing POC manufacturing as opposed to centralized manufacturing. While the centralized manufacturing system has a well-organized QA system, the challenge with decentralized system is that we may have facilities that are technically capable for its production but not experienced with the QA and regulatory aspects of GMP manufacturing. POC manufacturing necessitates a strong quality program at the site, particularly in the absence of quality program support from a commercial entity. For trials to move into the clinic, the manufacturing institution needs to possess professional knowledge of federal regulations and a robust team to handle information requests during the regulatory review process (39). For fresh CAR T products, a QA team that is knowledgeable and comfortable with the manufacturing process and QC tests is important to achieve a thorough but rapid batch record review and product release, as sampling, harvesting and distribution are typically performed over a 24 h time frame with a goal of infusing on first shift (68, 69). We summarize the important components required for setting up a GMP clean room in Table 4.

**Table 4:** Components of a GMP clean room.

1. Flush design and finish	The primary aim of a clean room is to minimize as few contaminants as possible. This is prioritized using a flush design for walls, windows, ceilings, and doors. It includes having no-edge windows, T-seals ceiling tiles, recessed light system, low air returns and flush finish sprinklers. GMP compliant doors need to be easily cleanable and resistant to cleaning agents. Sliding doors should be avoided.
2. Environmental and Microbiological monitoring	The monitoring system which includes temperature, humidity, and pressure ranges from $50,000–1 million dollars. The aim is to reduce microbial load, also known as bioburden. The design incorporates separate designated sections for specific activities such as a separate descrambling table, filling zone, stoppering zone, and a capping station.
3. Personnel and Material Air locks	Clean rooms must include personnel and material airlocks built between exit and entry points. The aim is to prevent microbial and particle contamination from protective gear.
4. Interlocking system and alarms	Interlocking and alarm systems are safe systems for opening and closing doors with minimum loss of pressure within the clean room. This prevents air exchange between the manufacturing area and the outside space.
5. At rest versus operation cleanliness level	Two separate levels of cleanliness need to be identified when workers are present within the room (*operation*) and when the clean room is unoccupied (*rest*). For example, maximum permitted number of particles at >0.5micron at rest is limited to 3,520–352,000 depending on the grade of operation.
6. HVAC system	The HVAC system plays an important role as it determines the number of air-changes required per hour depending on the dimension of the room and the products manufactured in the clean room.
7. Sinks and Drains	Sinks and drains should not be present in the clean room zone but are allowed in the gowning area. Mechanical valves should be installed between the sink and drains.

In the case of distributed, POC manufacturing, it would be incumbent upon the sponsor of the trial to oversee and continuously monitor (via audits and on-site visits) the operations and quality management at individual manufacturing sites. All documents would be subject to central review by the sponsoring company/entity. However, it is likely the quality agreement (which must be in place before manufacturing begins) would outsource real-time release testing results review and product sign-out to the POC manufacturing center, whose quality management system and leadership would have been thoroughly audited by the sponsor. Before trial initiation, technology transfer activities and verification studies would provide documentation of comparability in production and release testing methods across sites. Once the trial began, continuous communication and quality audits would protect against deviations and drift in critical quality attributes.

Financing POC manufacturing remains a challenge. Innovative approaches will be necessary to disseminate this approach. For example, the Biologics License Application FDA mechanism could allow a network of centers to produce CAR T cells, under the basic assumption that the process would be verifiable and similar in all production facilities, which would in turn market the product for sale. Another intriguing possibility is to seek institutional licensing similarly to what has been done with cord blood banks in the USA.

Toxicity profiles play an important role in the feasibility of in-house developed and manufactured products. Commercially available products have extensive documentation and a detailed spectrum of possible toxicities like cytokine release syndrome and neurotoxicity. It is expected that POC manufactured products will have such extensive documentation. Most importantly, if more than one center is to provide locally manufactured CAR T cells, all participating laboratories will have to show the same standard and comparable results, including similar release tests. The almost universal use of lymphodepleting agents like cyclophosphamide and fludarabine prior to the use of CAR T cells also significantly affects the toxicity profile. These potential responses require robust clinical and quality teams to monitor reactions.

## POC manufacturing of CART cells in the US system and global perspectives

Several academic institutions have started producing in-house products, like Case Western Reserve University Hospital (CWRU/UH) in Cleveland, Ohio, Medical College of Wisconsin (MCW) in Milwaukee, Wisconsin and Stanford, Palo Alto, California have utilized the CliniMACS platform using a lentiviral vector (13, 15, 32, 59). At CWRU/UH experience, 100% manufacturing success of anti-CD19 CAR T cells was achieved in 24 patients with median time from apheresis to infusion of 13 days. Similar efficacy and toxicity with other FDA approved CAR T cells products was observed (32, 59) At MCW and Stanford, POC manufacturing has been used in clinical trials of bispecific CAR T cells, including patients who had failed previous anti-CD19 CAR T cells, and they have shown feasibility with promising efficacy without safety signals concerns (13, 15) Twenty-six anti-CD19/CD20 CAR T cell products were manufactured using a fixed 14 days process in the CliniMACS Prodigy device. The target dose of CAR T cells was achieved in 85% of patients (22 of 26), with 100% successful manufacturing in CAR-naive patients (15) Similarly, the Ohio State University team is using the same platform to produce CD19/CD20/CD22 CAR T cells in 6–7 days to support a clinical trial (ClinicalTrials.gov Identifier: NCT05418088) (70).

Globally, production of CAR T cells is challenging. Different countries have different regulatory pathways, which adds to the challenge of having a single uniform international regulatory standard. Despite this, some countries have developed innovative approaches for POC production.

For instance, in Spain, a national network of hospitals was established for production of CD19-specific CAR T cells used in the treatment of relapsed ALL that resulted in approval by the European Medicines Agency (EMA). On the Spanish group pivotal clinical trial, 54 patients underwent apheresis with 87% of success in CAR T cell manufacture. The median vein-to-vein time was not yet shorter than centralized manufactured products, mostly due to 7 patients who required 2 apheresis and numerous intervening medical complications that forced delays to start lymphodepleting chemotherapy and cell infusion (38).

In China, authorities have encouraged small start-up companies to produce these products in direct collaboration with a clinical center (71). Through this partnership and decentralized CAR T cells manufacture, there are over 500 Chinese clinical trials registered aiming to improve CAR T cells manufacture efficiency and efficacy. An example is provided by Nanjing Biolegend with its anti-BCMA CAR T cell for multiple myeloma, now approved by FDA and promising data from early phase clinical trials with anti-claudin CAR T cells in gastrointestinal malignancies (4, 18, 71).

Similarly centers in Germany, Israel, India, and Brazil have started introducing the concept of POC production, with some clear challenges remaining to full access. For instance, Palani et al. in India have demonstrated production of CAR T cells using the ClinicMACS platform, but no patients have been treated due to the limitation of financial assistance. All the above centers have laid foundation for middle income countries to begin discussions about setting up research labs, thereby enhancing the global acceptance of this novel product (24, 30, 72).

## Intellectual property, research & development investment, and equitable access

Early-stage CAR T cell products were initially funded by government institutions or philanthropic societies. The next generation of CAR T products should reach all countries, regardless of national income or funding abilities. As mentioned by Lam and collaborators in the United Kingdom, decentralized manufacturing can be quite cost effective, particularly if multiple smaller units are set up in a common geographical area, thereby allowing equipment and personnel to be better utilized at a local level (29). Sharing resources between multiple hospitals could benefit a larger population by easy accessibility to these products. Likewise, partnerships with pharmaceutical suppliers would more readily facilitate hospital-based studies and clinical trials for production of genetically modified products in a cost-effective manner. Indeed, biotech companies are beginning to appreciate the advantages of POC manufacturing and are working to roll out their manufacturing and testing platforms to select academic centers with the goal of commercializing with regional manufacturing centers around the world (15, 32, 38).

## Healthcare legislation

Healthcare legislation underpins all modern research and technology. To expand the use of cell and gene therapies across the world, especially in low-income areas, a uniform code of practice should be formulated to ensure adequate quality of production of these products in a safe environment. Legislation is necessary to ensure protection from unethical and harmful practices, particularly when such regulations have not been codified before. An individual's right to permit the use of his or her own cells must always be protected. The adoption of the WHO concept of Universal Health Coverage (UHC) where “all communities can use promotive, preventive, curative and rehabilitative services they need while ensuring that the use of these services does not expose the user to financial hardships” is an essential step in expanding therapeutic access and enabling high-quality research (73).

## Health economics and capacity building

Given the high cost of production and delivery limiting therapeutic use in various countries, resource allocation will play an essential role in implementing a therapeutic program in emerging markets. An important concept of the ICER, or incremental cost-effective ratio, serves as a basis for considering an intervention as good value for money. Using this concept, various programs in Africa for treating hemophilia and providing cancer chemotherapy have reached a wider network of patients (74) This concept can be easily applied to cell and gene therapy by implementing POC manufacturing in targeted geographical areas and appropriate demographic areas that will provide maximum benefits for patients (75).

Current CAR T cell product prices ranges anywhere between USD $373,000 to $475,000 which is unaffordable in most nations (29, 30, 76) The use of decentralized production will be beneficial in such scenarios where pharmaceutical production will not be profitable. Also, the use of partnerships between low- and medium-income nations mat achieve a balance between cost-effectiveness and affordability. An example of this partnership is seen between South Africa and India where two biotech companies are providing services of their genetic therapies at a tenfold lower price (74, 76).

Capacity building mainly includes strengthening the workforce. The administration and production of these products need a strong local workforce. Collaborations between different countries mainly for training and education is extremely important. Various universities have increased scholarships for students from low-income nations. An initiative started by the South-African government is the BM-NHSP (Bongani Mayosi National Health Scholars Program) where students are trained in genetic and cellular therapy (74). A similar initiative was started by the Bill & Melinda Gates Foundation with the NIH (National Institutes of Health) that supports genetic therapy in under-resourced nations. To fully capitalize on decentralization, training of skilled workers in high-technology equipment production and maintenance at a local level is essential to ensure high quality therapeutic use and patient care. As enumerated above, these modifications will help in maintaining a high quality of research worldwide and will help countries reap the benefits of genetically modified agents that were once considered unavailable to them.

## Conclusion

POC manufacturing is a promising approach to expand the availability and utilization of CAR T cell therapies worldwide in a both time- and cost-effective manner. While there are several obstacles to overcome (e.g., availability of vectors, personnel training, lack of facilities), several initiatives are underway to address these issues. In addition, stringent regulatory oversight is required to maintain the quality and reproducibility of these products worldwide. Likewise, academic-pharmaceutical partnerships will be necessary to promote future research and innovation in the field.
